# First record of a neonate bluntnose sixgill shark (*Hexanchus griseus*) from Baja California Sur, Mexico

**DOI:** 10.1002/ece3.11664

**Published:** 2024-06-30

**Authors:** Darren A. Whitehead, Joel H. Gayford

**Affiliations:** ^1^ Investigación Tiburones Mexico A.C La Paz Mexico; ^2^ Department of Life Sciences Imperial College London London UK; ^3^ Shark Measurements London UK; ^4^ College of Science and Technology James Cook University Townsville, QLD Australia

**Keywords:** hexanchiformes, Mexican Pacific, nursery area, pupping ground, reproductive periodicity

## Abstract

The bluntnose sixgill shark (*Hexanchus griseus*) is a wide‐ranged deep‐water shark species found off continental and insular shelves. Despite its global distribution, little is known about the reproductive ecology of the species, particularly with regard to the location and timing of important phenological events such as mating and pupping. In this study, we report the landing of a neonate *H. griseus* individual from an artisanal fishing camp in Baja California Sur, Mexico. This represents only the ninth confirmed record of the species from the Mexican Pacific and the first to report a neonate specimen in Mexican waters. We discuss this specimen in the context of the environmental conditions in which it was found, ultimately suggesting that these shallow coastal waters may be an important pupping ground for *H. griseus* in the region. Furthermore, the specimen was found during the winter months (whereas all previous reports have suggested *H. griseus* pups during the summer), implying regional variation in reproductive periodicity, or the presence of multiple reproductive events per year. This study provides novel insight into the reproductive biology of *H. griseus* and the ecological characteristics of the species in the Northern Mexican Pacific.

## Graphical abstract

We provide a new record of a neonate sixgill shark from Baja California Sur, Mexico. The location and timing of this specimen provide important novel insight into the spatial and reproductive ecology of the species, both in the Mexican Pacific and more broadly.

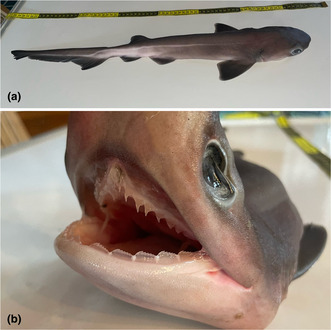

## 1 INTRODUCTION

Sharks and rays (Elasmobranchii) are a critically important component of marine ecosystems, fulfilling diverse ecological niches as apex and meso‐predators amongst other roles (Heithaus et al., 2010; Roff et al., 2016). Despite their ecological importance and taxonomic diversity, overfishing, amongst other threats, has driven over one‐third of elasmobranch species towards extinction (Dulvy et al., 2021). One of the major limitations affecting our ability to conserve and protect elasmobranch species successfully is data deficiency – with over 150 species classed as data deficient by the international union for the conservation of nature (IUCN) in 2021 (Dulvy et al., 2021). This data deficiency implies a lack of basic biological/ecological information, including distribution, abundance and life‐history traits (Leurs et al., 2023; Walls & Dulvy, 2020). In light of this issue and the dire conservation status of many elasmobranch populations (Dulvy et al., 2021; Pacoureau et al., 2021), improving our baseline ecological and biological knowledge of shark species across their distribution is of utmost importance.

The bluntnose sixgill shark (*Hexanchus griseus*) is a large‐bodied hexanchiform shark distributed globally across continental and insular shelf and slope environments (Ebert et al., 2021). *Hexanchus griseus* occupies a wide depth range, as it has been observed by divers in shallow waters around 40 m and by submersibles at depths greater than 2500 m (Compagno, 1984; Ebert et al., 2021). An economically important species, *H. griseus* is the focus of SCUBA dive tourism in the United States and Canada (Dunbrack, 2008; Ebert et al., 2021; Healy et al., 2020) and has formed a component of catches within deep‐sea fisheries (Ebert, 1986; Mili et al., 2021; Nuez et al., 2023). Unfortunately, *H. griseus* is vulnerable to overfishing and has become scarce across parts of its range as a result (Anderson et al., 1999; Ebert et al., 2021). Whilst not listed as data deficient by the IUCN (Finucci et al., 2020), little is known about the biology and ecology of *H. griseus* across much of its range. In some cases, knowledge of the species in each region comes from just a handful of studies focused on a few individuals (Becerril‐García et al., 2017; Lipej et al., 2022). In the Mexican Pacific, for example, only 8 records of the species exist in the literature (Becerril‐García et al., 2017). Without further study, it is impossible to discern the status of *H. griseus* populations, the extent to which they may be threatened by intense fishing pressure and to provide the necessary management and protection measures to prevent population decline (Vella & Vella, 2017).

In this study, we provide a new report of a neonatal *H. griseus* individual landed in an artisanal fish camp in Baja California Sur, Mexico, including morphometric measurements. The context within which the individual was caught provided new insight into the ecology of the species and its distribution within the region. In this light, we suggest that the region may represent a nursery and/or pupping ground for *H. griseus*, and thus an important area of interest for future research and conservation of the species. We also suggest that reproductive seasonality in this population may differ from that reported in other studies. This represents just the 9th confirmed record of *H. griseus* from the Mexican Pacific and the smallest to date. Ascertaining biological information about this species is critical given the high levels of shark meat consumption in the region (Ojeda‐Ruiz et al., 2018; Smith et al., 2009) and almost complete lack of knowledge regarding the ecology and population status of *H. griseus* in Mexican waters.

## 2 METHODOLOGY

**FIGURE. 1:**
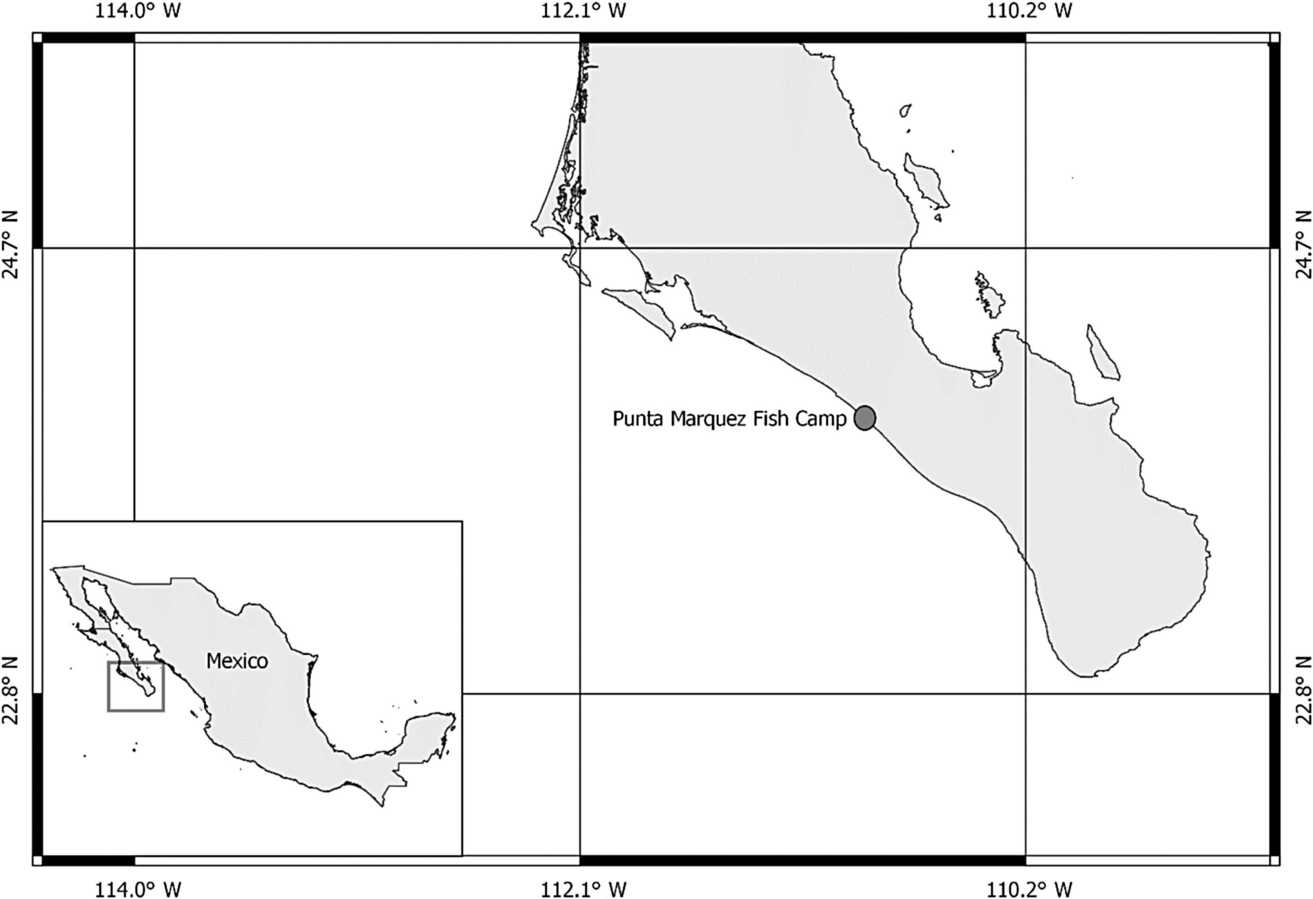
Showing artisanal fish camp where the recovered shark was landed.

The specimen was caught on 3 February, 2023 at a fish camp located at Punta Marquez 36 km north of the city of La Paz (23°57′33.6″ N 110°52′35.9″ W, Figure 1). A small hexanchiform shark was landed by artisanal fishermen and given to researchers for identification and measurement. The specimen was caught using standard gill nets approximately 500 m from the shore at approximately 45 m depth. Upon retrieval of the catch, fishermen reported that a second individual of the same species (and of approximately equivalent size) was found; however, it escaped alive before the net could be fully pulled aboard. Following recovery of the shark from the camp, it was transported to a laboratory for analysis. The specimen was examined to determine species, sex and ontogenetic stage, and a series of morphological measurements were taken (see Gayford et al., 2023 for a detailed description of these measurements).

## 3 RESULTS

**FIGURE 2:**
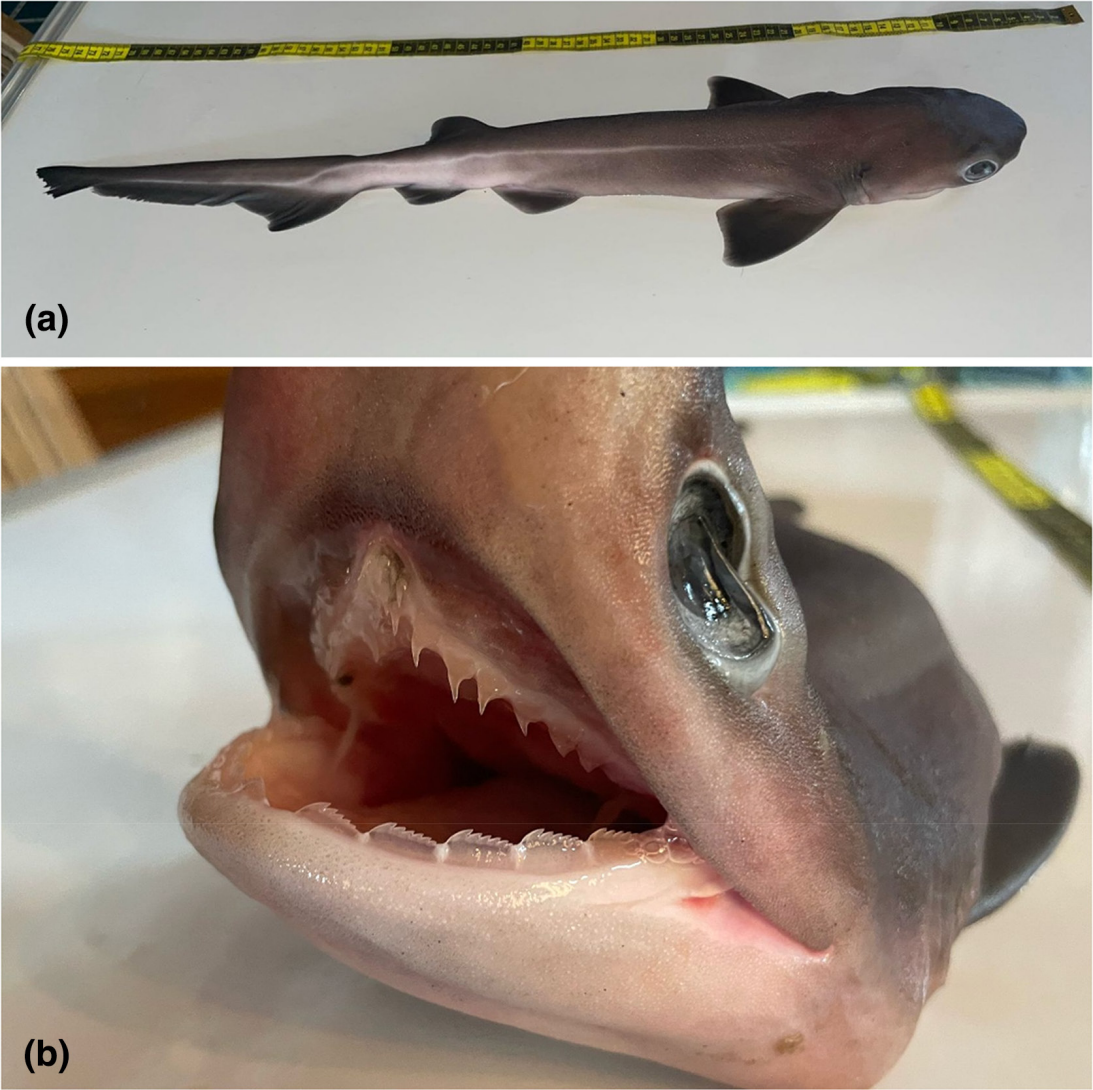
Photographs showing morphological features of the specimen from lateral (a) and frontal (b) views. Note the characteristic hexanchiform dentition visible in (b).

The specimen was identified as belonging to the species *Hexanchus griseus* based on external morphological features, including a subterminal mouth, six pairs of gill slits and a round, blunted snout (Figure 2; Ebert et al., 2021). This is not surprising given that *H. griseus* is the only sixgill shark species known to inhabit the Mexican Pacific (Becerril‐García et al., 2017). Due to the absence of claspers, the specimen was determined as a female. Additionally, a total length of 63 cm (Table 1) suggests that the specimen is a neonate, as the species is thought to be born at a total length of 61–74 cm, and due to the presence of an umbilical scar (Ebert et al., 2021). The artisanal fishermen who collected the specimen reported that large individuals of the species (>2 m total length) are caught seasonally in the area, at similar depth ranges and using similar fishing equipment to that in which the neonate specimen was found.

**TABLE 1:** Morphological measurements extracted from the neonate *Hexanchus griseus* specimen.

Measurement	Length (cm)
Total length (TL)	63.0
Fork length (FL)	46.3
Upper caudal length (UL)	22.0
Lower caudal length (LL)	6.0
Caudal height (CH)	19.5
Caudal keel diameter (CK)	7.0
Posterior span (PS)	10.5
Frontal span (FS)	10.5
Lateral span (LS)	11.5
Eye‐to‐eye length (EE)	7.0
Pectoral fin length (PL)	9.0
Dorsal fin length (DL)	5.5
Dorsal fin height (DH)	2.5
Dorsal fin width (DW)	6.0

*Note*: See Gayford et al. (2023) for detailed definitions of these measurements.

## 4 DISCUSSION

The specimen described in this study represents the 9th record of *Hexanchus griseus* from the Mexican Pacific and the smallest and only neonate to be documented in the region. Little is known about basic ecology characteristics of the species across much of its range (Rodríguez‐Cabello et al., 2018), with a complete lack of any such data from the Mexican Pacific. This specimen, placed in the ecological context in which it was caught, provides novel insight into the ecology of *H. griseus* in the region, which may in turn facilitate further study and contribute to conservation and protection of the species.

The small size of the specimen described here points towards the coastal waters off Punta Marquez, Baja California Sur, as a nursery and/or potential pupping ground for *H. griseus*. Based on the morphological measurements taken from the specimen (Table 1), it is undoubtedly a neonate (Ebert et al., 2021). Juvenile *H. griseus* are known to inhabit shallower waters than their older conspecifics, with neonates in particular favouring coastal areas (Compagno, 1984; Desbrosses, 1938; Rodríguez‐Cabello et al., 2018). As a deep‐water species, adults spend the majority of their time in the cold waters below the thermocline (Comfort & Weng, 2015; Rodríguez‐Cabello et al., 2018), although they have been known to exhibit vertical movements through the water column, potentially associated with foraging behaviour (Comfort & Weng, 2015). Information about the reproductive biology and reproductive behaviour of *H. griseus* is scarce; however, some authors have suggested that pupping occurs on upper slopes and outer continental shelves (Comfort & Weng, 2015; Ebert, 2002). The finding of a neonate *H. griseus* in shallow coastal waters suggests in itself that this coastal zone is likely in close proximity to nursery grounds for the species. However, combined with the reported seasonal presence of adult individuals in the artisanal fishery, this points towards an active pupping ground for the species. Interestingly this suggests that pupping may occur closer to shore and in shallower waters than previously hypothesised (Comfort & Weng, 2015; Ebert, 2002). We cannot conclusively prove that this location represents a regular pupping ground for the species, but based on the small size of the specimen we have described, and the first‐hand report of a second individual that escaped capture on that day, the most parsimonious explanation is that these individuals were born in close proximity to the site of capture. Similar conclusions have been drawn from fisheries records of neonate *H. griseus* individuals from both Africa and Europe (Desbrosses, 1938; Ebert, 2002).

It is not only the location of pupping in *H. griseus* that is poorly understood; the reproductive periodicity of the species is unstudied. Most shark species show some degree of reproductive seasonality, with mating and pupping occurring during discrete periods of time that may coincide with favourable environmental conditions and/or seasonal prey abundance (Awruch et al., 2009; Carrier et al., 2004; Whitney et al., 2012). Whilst no study has directly assessed reproductive seasonality in *H. griseus*, neonates are typically found in shallow waters during the summer months, in both the northern and southern hemispheres (Desbrosses, 1938; Ebert, 2002). This neonate was landed during the Northern Hemisphere winter, however, suggesting either the presence of multiple reproductive cycles per year, or significant regional variation in reproductive seasonality. In either case, further clarifying reproductive seasonality in *H. griseus* and the extent to which it differs between populations will be key to ensuring the species' proper management and conservation.

Considering this specimen and additional reports of *H. griseus* in close vicinity of the Punta Marquez camp fishing area, further research is warranted. Reports of additional neonates and adult females from this area may strengthen our claim of a possible pupping zone, particularly if active pregnancy in adult females can be confirmed. In addition, spatial ecological studies of the population utilising tagging technology (e.g. Andrews et al., 2010) may help reveal additional details about the habitat usage of *H. griseus* in the Mexican Pacific. Novel records of neonate *H. griseus* individuals may enable us to discern the reproductive periodicity of the species, as the information presented in this study raises doubts about a summer reproductive season at least within populations in the Mexican Pacific. Given the scarcity of previous records of the species from the Mexican Pacific (Becerril‐García et al., 2017), it may be difficult to alleviate any of these research gaps or discern population status using traditional ecological techniques. We suggest that in this case, the use of emerging eDNA technologies as applied to other ‘rare’ or elusive elasmobranch populations (Boussarie et al., 2018; Ip et al., 2021; Pikitch, 2018) may prove highly beneficial for studying the habitat usage, distribution and abundance of *H. griseus* in the region.

## 5 CONCLUSIONS

This finding of a neonate *H. griseus* individual in the shallow coastal waters of the Pacific near to Punta Marquez suggests that this area may be important for the management and conservation of the species in Mexican waters. Combined with the particularly small size of the specimen and reports of adult specimens caught in a similar location, we tentatively suggest that this may represent a pupping ground for *H. griseus*. Intriguingly, the temporal context within which this specimen was caught suggests that reproductive seasonality in *H. griseus* may differ between populations, and that in the Mexican Pacific, pupping either occurs during winter or occurs more than once per year. Considering these findings, additional research is required to improve our understanding of the reproductive biology of *H. griseus* on a global scale, and the ecology and population status of the species in the Mexican Pacific.

## AUTHOR CONTRIBUTIONS

**Darren A. Whitehead:** Conceptualization (equal); data curation (lead); methodology (equal); writing – original draft (equal); writing – review and editing (equal). **Joel H. Gayford:** Conceptualization (equal); methodology (equal); writing – original draft (equal); writing – review and editing (equal).

## FUNDING INFORMATION

The authors report no funding for this study.

## CONFLICT OF INTEREST STATEMENT

The authors declare no conflicts of interest regarding this study.

## Data Availability

All datasets generated during this study are available within the article.
